# Chemical and Synthetic Genetic Array Analysis Identifies Genes that Suppress Xylose Utilization and Fermentation in *Saccharomyces cerevisiae*

**DOI:** 10.1534/g3.111.000695

**Published:** 2011-09-01

**Authors:** Jane Usher, Victor Balderas-Hernandez, Peter Quon, Nicholas D. Gold, Vincent J. J. Martin, Radhakrishnan Mahadevan, Kristin Baetz

**Affiliations:** * Ottawa Institute of Systems Biology, Department of Biochemistry, Microbiology and Immunology, University of Ottawa, Ottawa, Ontario, Canada K1H 8M5; † Department of Chemical Engineering and Applied Chemistry, Institute of Biomaterials and Biomedical Engineering, University of Toronto, Toronto, Ontario, Canada M5S 3E5, and; ‡ Department of Biology, Concordia University, Montréal, Québec, Canada H4B 1R6

**Keywords:** recombinant yeast, ethanol, xylose, functional genomics, chemical genomics

## Abstract

Though highly efficient at fermenting hexose sugars, *Saccharomyces cerevisiae* has limited ability to ferment five-carbon sugars. As a significant portion of sugars found in cellulosic biomass is the five-carbon sugar xylose, *S. cerevisiae* must be engineered to metabolize pentose sugars, commonly by the addition of exogenous genes from xylose fermenting fungi. However, these recombinant strains grow poorly on xylose and require further improvement through rational engineering or evolutionary adaptation. To identify unknown genes that contribute to improved xylose fermentation in these recombinant *S. cerevisiae*, we performed genome-wide synthetic interaction screens to identify deletion mutants that impact xylose utilization of strains expressing the xylose isomerase gene *XYLA* from *Piromyces sp*. E2 alone or with an additional copy of the endogenous xylulokinase gene *XKS1*. We also screened the deletion mutant array to identify mutants whose growth is affected by xylose. Our genetic network reveals that more than 80 nonessential genes from a diverse range of cellular processes impact xylose utilization. Surprisingly, we identified four genes, *ALP1*, *ISC1*, *RPL20B*, and *BUD21*, that when individually deleted improved xylose utilization of both *S. cerevisiae* S288C and CEN.PK strains. We further characterized *BUD21* deletion mutant cells in batch fermentations and found that they produce ethanol even the absence of exogenous *XYLA*. We have demonstrated that the ability of laboratory strains of *S. cerevisiae* to utilize xylose as a sole carbon source is suppressed, which implies that *S. cerevisiae* may not require the addition of exogenous genes for efficient xylose fermentation.

Cellulosic fermentation for the production of fuels and chemicals has many advantages as cellulose, the main component of plant cell walls, is very abundant in agricultural and forestry waste and this feedstock does not compete with valuable food source (reviewed in Lynd *et al.* 2008; Rubin 2008). Though cellulose is abundant and renewable, numerous hurdles remain in making cellulosic ethanol production an economically viable industry. In contrast to cane sugar or starch fermentation, due to the complex nature of the carbohydrate present in cellulosic biomass, a significant amount of xylose and arabinose (5-carbon sugars derived from the hemicellulose portion of the lignocellulose) is present in the biomass hydrolysates (Saha 2003). Indeed, after glucose, D-xylose is the second most abundant sugar in hemicelluloses. Therefore, in order to maximize the potential for ethanol production, ethanologenic fermentation strains must be capable of utilizing both pentose and hexose sugars present in the lignocellulose.

Though *Saccharomyces cerevisiae* has an exceptional ability for rapid anaerobic growth and fermentation of hexose sugars, it has been generally reported that laboratory strains exhibit only a negligible metabolism of xylose (Chiang *et al.* 1981; Gong *et al.* 1983; Wang *et al.* 1980). This phenotype is in spite of the fact that S288C laboratory yeast has endogenous genes that appear to encode a putative xylose utilization pathway (Figure 1). The present strategy to improve the ability of *S. cerevisiae* to ferment xylose has been the introduction of exogenous genes from xylose-fermenting fungi (reviewed in Hahn-Hagerdal *et al.* 2007; Matsushika *et al.* 2009; Van Vleet and Jeffries 2009). The most common strategy involves the introduction of xylose reductase (XR) and xylitol dehydrogenase (XDH) genes and the overexpression of the endogenous xylulokinase gene (*XKS1*) (Hallborn *et al.* 1991; Ho *et al.* 1998; Jin *et al.* 2002; Kotter *et al.* 1990). As the XR/XDH pathway can result in a cofactor imbalance that has been shown to negatively impact metabolic flux (reviewed in Hahn-Hagerdal *et al.* 2007; Matsushika *et al.* 2009; Van Vleet and Jeffries 2009), a second strategy has emerged that introduces a bacterial xylose isomerase (XI) gene into yeast, which allows for the slow metabolism of xylose via the endogenousXks1 (Karhumaa *et al.* 2005; Kuyper *et al.* 2003; Madhavan *et al.* 2009; Walfridsson *et al.* 1996). Considerable effort has been made to improve pentose fermentation, including engineering or optimizing xylose enzyme activity (XR, XK, XDH, and XI), xylose transport, and the pentose phosphate pathway; reducing redox imbalances; and other strategies (reviewed in Hahn-Hagerdal *et al.* 2007; Matsushika *et al.* 2009). Despite significant directed efforts focusing on known proteins or pathways impacting xylose utilization and fermentation, ethanol production from xylose remains inefficient in recombinant *S. cerevisiae* strains and suggests that novel strategies should be considered.

**Figure 1:**
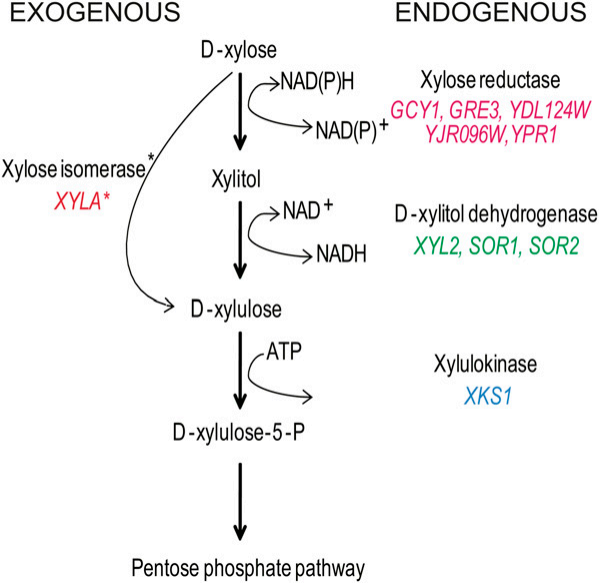
The xylose utilization pathway in *S. cerevisiae*, including xylose isomerase strategy to improve xylose utilization. Putative endogenous *S. cerevisiae* genes of the xylose utilization pathway are listed on the right and marked in different colors: pink for genes involved in xylose reducatase pathways; green for xylitol dehydrogenase; and blue for xylulokinase. The exogenous xylose isomearse gene *XYLA* is listed on the left, highlighted in red, and marked by *. For this study, *XYLA* was isolated from *Piromyces Sp E2*. Adapted from Wenger *et al.* (2010).

It is clear that despite the common belief that *S. cerevisiae* cannot use xylose as a carbon source, many wild and industrial wine yeast strains are in fact capable of xylose utilization (Attfield and Bell 2006; Wenger *et al.* 2010). The ability of some industrial yeast to utilize xylose was recently mapped to a putative xylitol dehydrogenase gene, *XDH1* that is not present in the laboratory S288C strain (Wenger *et al.* 2010). Importantly, Wenger *et al.* (2010) determined that *XDH1* requires the endogenous XR genes *GRE3* and *YPR1* and the endogenous XK gene *XKS1* to allow for xylose utilization. In contrast, they also found that three putative XDH genes, *SOR1*, *SOR2*, and *XYL2*, suppressed the ability of *XDH1* expressing strains to utilize xylose (Wenger *et al.* 2010). This work not only illustrated that *S. cerevisiae* is genetically “primed” to ferment xylose but also suggested that additional endogenous proteins may either positively or negatively impact the ability of *S. cerevisiae* to utilize xylose. In an attempt to isolate such proteins, we performed genome-wide synthetic genetic array (SGA) screens (Tong *et al.* 2001) to identify deletion mutants that impact the xylose utilization of strains expressing the XI gene *XYLA* from *Piromyces sp*. E2 (herein referred to as *pXYLA*) and strains expressing both *XYLA* and an additional copy of the endogenous XK gene *XKS1* (herein referred to as *pXYLA,XKS1*). To compliment this effort, we screened the deletion mutant array to identify mutants whose growth is either negatively or positively impacted by xylose. Our genetic network reveals that more than 80 nonessential genes from a diverse range of cellular processes impact xylose utilization. Surprisingly, we identified four deletion mutants that improved xylose consumption of laboratory S288C, even in the absence of exogenous *XYLA*. Next, we deleted these genes in an ethanol-tolerant CEN.PK strain and confirmed that all four mutants improved xylose utilization and that the deletion of *BUD21* improves xylose fermentation. These data suggest that the natural ability of *S. cerevisiae* to recognize and utilize xylose was suppressed, and they validate the use of systems biology approaches using S288C as a means to identify novel pathways contributing to xylose fermentation.

## Materials and Methods

### Yeast strains

The yeast strains used in this study are listed in Table 1. The *MAT****a*** deletion mutant array (DMA) was purchased from OpenBiosystems (Catalog no. YSC1053). The deletion strains generated for this study were designed using a standard PCR-mediated gene insertion technique (Longtine *et al.* 1998) and confirmed by PCR analysis using two sets of primer pairs (sequences available upon request). Plasmids were transformed into wild-type cells using a standard LiAc protocol (Gietz and Schiestl 2007).

**Table 1:** *S. cerevisiae* strains used in this study

Strain Name	Genetic Background	Origin
Y7092	*MAT***α** *can1Δ*::*STE2pr-SP-his5 lyp1Δ his3Δ1 leu2Δ0 ura3Δ0 met15Δ0 LYS2*+	Tong and Boone 2006
YKB2179	*MAT***α** *can1Δ*::*STE2pr-SP-his5 lyp1Δ his3Δ1 leu2Δ0 ura3Δ0 met15Δ0 LYS2+ pXYLA*	This study
YKB2178	*MAT***α** *can1Δ*::*STE2pr-SP-his5 lyp1Δ his3Δ1 leu2Δ0 ura3Δ0 met15Δ0 LYS2+ pXYLA,XKS1*	This study
YKB2069	*MAT***α** *can1Δ*::*STE2pr-SP-his5 lyp1Δ his3Δ1 leu2Δ0 ura3Δ0 met15Δ0 LYS2+alp1*Δ::*kanMX6*	This study
YKB2530	*MAT***α** *can1Δ*::*STE2pr-SP-his5 lyp1Δ his3Δ1 leu2Δ0 ura3Δ0 met15Δ0 LYS2+ isc1*Δ::*kanMX6*	This study
YKB2531	*MAT***α** *can1Δ*::*STE2pr-SP-his5 lyp1Δ his3Δ1 leu2Δ0 ura3Δ0 met15Δ0 LYS2+ rpl20b*Δ::*kanMX6*	This study
YKB2532	*MAT***α** *can1Δ*::*STE2pr-SP-his5 lyp1Δ his3Δ1 leu2Δ0 ura3Δ0 met15Δ0 LYS2+ bud21*Δ::*kanMX6*	This study
YKB2534	*MAT***α** *can1Δ*::*STE2pr-SP-his5 lyp1Δ his3Δ1 leu2Δ0 ura3Δ0 met15Δ0 LYS2+ alp1*Δ::*kanMX6 isc1*Δ::*NAT*	This study
YKB2535	*MAT***α** *can1Δ*::*STE2pr-SP-his5 lyp1Δ his3Δ1 leu2Δ0 ura3Δ0 met15Δ0 LYS2+ alp1*Δ::*kanMX6 rpl20b*Δ::*NAT*	This study
YKB2536	*MAT***α** *can1Δ*::*STE2pr-SP-his5 lyp1Δ his3Δ1 leu2Δ0 ura3Δ0 met15Δ0 LYS2+ isc1Δ*::*kanMX6 rpl20b*Δ::*NAT*	This study
YKB2537	*MAT***α** *can1Δ*::*STE2pr-SP-his5 lyp1Δ his3Δ1 leu2Δ0 ura3Δ0 met15Δ0 LYS2+ bud21Δ*::*kanMX6 alp1Δ*::*NAT*	This study
YKB2538	*MAT***α** *can1Δ*::*STE2pr-SP-his5 lyp1Δ his3Δ1 leu2Δ0 ura3Δ0 met15Δ0 LYS2+ bud21Δ*::*kanMX6*, *isc1Δ*::*NAT*	This study
YKB2539	*MAT***α** *can1Δ*::*STE2pr-SP-his5 lyp1Δ his3Δ1 leu2Δ0 ura3Δ0 met15Δ0 LYS2+ bud21Δ*::*kanMX6 rpl20bΔ*::*NAT*	This study
YKB2684	*MAT***a** *ura3-52 trp1-289 leu2-3,112 his3*Δ*1 MAL2-8C SUC2* (CEN.PK 113-13D)	Entian and Koetter 1998
YKB2680	*MAT***a** *ura3-52 trp1-289 leu2-3,112 his3*Δ*1 MAL2-8C SUC2 + pXYLA,XKS1*	This study
YKB2666	*MAT***a** *ura3-52 trp1-289 leu2-3,112 his3*Δ*1 MAL2-8C SUC2 bud21*Δ:*:kanMX6*	This study
YKB2667	*MAT***a** *ura3-52 trp1-289 leu2-3,112 his3*Δ*1 MAL2-8C SUC2 bud21*Δ::*kanMX6 + pXYLA,XKS1*	This study
YKB2668	*MAT***a** *ura3-52 trp1-289 leu2-3,112 his3*Δ*1 MAL2-8C SUC2 alp1Δ*::*kanMX6*	This study
YKB2670	*MAT***a** *ura3-52 trp1-289 leu2-3,112 his3*Δ*1 MAL2-8C SUC2 isc1Δ*::*kanMX6*	This study
YKB2665	*MAT***a** *ura3-52 trp1-289 leu2-3,112 his3*Δ*1 MAL2-8C SUC2 rpl20bΔ*::*kanMX6*	This study

### Plasmid construction for xylose isomerase (*XYLA*) and xylulokinase (*XKS1*)

The *XYLA* and *XKS1* genes were cloned into the pYES2 shuttle vector (Invitrogen), each preceded by the Kozak sequence (GCCACC) and flanked by the triosephosphate isomerase constitutive promoter (*TPI*p) and thecytochrome C1 transcription terminator (*CYC1*tt). First, the *GAL1* promoter sequence was removed from pYES2, and then *TPI*p and *XYLA* were amplified by PCR with genomic DNA from *S. cerevisiae* and *Piromyces* sp. E2 genomic DNA, respectively, and inserted upstream of *CYC1*tt, as described previously (Kuyper *et al.* 2003), resulting in pYES2-*XYLA-CYC1*tt [herein called *pXYLA* (pKB60)]. *TPIp* was obtained using the primer combination of TPI-*Nhe*I-Fwd 5′-GATCGCTAGCTGTTsTAAAGATTACGGATAT-3′ and TPI-*Eco*RI-Rev 5′-GATCGAATTCTTTTAGTTTATGTATGTGTTTTTTGTAG-3′. Primers for PCR amplification from *S. cerevisiae* were based on genomic sequence data for strain S288C, although the strain used as DNA template was CEN.PK113-13D. Phusion HF DNA polymerase (Finnzymes) was used for all PCR reactions. Next, a *XKS1-CYC1*tt-*TPI*p cassette was assembled. A second *TPI*p was amplified by PCR using primer pair TPI-*Hin*dIII-Fwd 5′-GATCAAGCTTTGTTTAAAGATTACGGATAT-3′ and TPI-*Eco*RI-Rev. *CYC1*tt could not be successfully PCR-amplified from the pYES2 vector, so the following strategy was taken to excise it for cloning: pYES2 was digested with *NsiI* and religated upon itself, reducing it to a 2.2-kbp mini-pYES2 version of itself, still containing the *bla* resistance gene *CYC1*tt with a single *NsiI* site just upstream of it and the pUC origin. A *HindIII* site was introduced 100 bp downstream of *CYC1*tt by PCR-amplifying mini-pYES2 with the primer combination of pYES-CYC-*Avr*II-Fwd 5′-GATCCCTAGGTTCGGCTGCGGCGAGCGGTA-3′ and pYES-CYC-*Hin*dIII-*Avr*II-Rev 5′-GATCCCTAGGAAGCTTCGACCGAGCGCAGCGAGTCA-3′. The resulting PCR product was digested with *AvrII*, ligated upon itself, and then transformed into *Escherichia coli* DH5α. The vector was reisolated and digested with *NsiI* and *HindIII* to release the *CYC1*tt fragment. *XKS1* was amplified by PCR from *S. cerevisiae* genomic DNA with primer pairXKS1-*Eco*RI-Fwd 5′-GATCGAATTCGCCACCATGAGAGTCTTTTCCAGTTCGCTTAA-3′ andXKS1-*Nsi*I-Rev 5′-GATCATGCATATGTTGTGTTCAGTAATTCAGAGACAG-3′. The *XKS1* and new *TPIp* PCR products were digested with *NsiI* and *HindIII*, respectively, and in a single reaction ligated together with the *CYC1*tt fragment. The resulting cassette was reamplified by PCR using primersXKS1-*Eco*RI-Fwd and TPI-*Eco*RI-Rev, digested with *EcoRI*, and inserted at the *EcoRI* site between the first *TPI*p and *XYLA* in pYES2-*TPI*p-*xylA-CYC1*tt, resulting in the final pYES2-*TPI*p-*XKS1-CYC*tt-*TPI*p-*XYLA-CYC1*tt vector [herein called *pXYLA,XKS1* (pKB61)].

### Growth conditions and dot assay experiments

Cells were grown in standard YEP or synthetic complete (SC) media supplemented with glucose or xylose to a final concentration of 2%, unless otherwise described. To assess growth under xylose and glucose conditions, wild-type cells were grown in YEP media supplemented with either 2% glucose or xylose, and transformed cells were grown in SC-uracil (to maintain the plasmid) supplemented with either 2% xylose or glucose. Cells were grown to midlog phase in the carbon source of interest, and then either growth curve or dot assays were performed. Semi-aerobic growth curve analysis was performed in triplicate on Multiskan Ascent plate reader (Thermo Electron Corporation) in sealed plates, at 30° with shaking at 480 rpm prior to the OD_600_ measurements that were taken every 30 min over a 20-hr period. Dot assays were performed by spotting 5 µl of 10-fold serial dilutions (OD600 = 0.1, 0.01, 0.001, 0.0001) onto specified media, and sealed plates were incubated at 30°. All growth curve and dot assay experiments were repeated using three different isolates of each strain.

### Glucose and xylose consumption assays

Independent yeast colonies were cultured in 2 ml of selective media supplemented with either 2% glucose or xylose and incubated at 30° overnight with shaking. Following overnight culturing, 500 µl of the culture was transferred into 10 ml of fresh media supplemented with 2% xylose or glucose and incubated for 24 hr at 30°. Cells were harvested by centrifugation, washed once with sterile YEP media, and pitched at 1.5 × 10^7^ cells/ml into shaker flasks containing either 100 ml of glucose- or xylose-containing media at 13 g/l. Flasks were shaken at 250 rpm at 30°. Consumption assays were performed in triplicate, and samples were taken at 24-hr intervals to check for sugar utilization. The glucose concentration was determined using the Glucose (GO) Assay Kit (Sigma GAGO-20) per the manufacturer’s guidelines. Xylose concentration was determined using the phloroglucinol assay (Eberts *et al.* 1979). Briefly, the color reagent of 0.5 g of phloroglucinol (Sigma), 100 ml of glacial acetic acid, and 10 ml of concentrated HCl was freshly prepared and kept in the dark. Stock standard xylose (10 g/l) was prepared by dissolving D-xylose powder in saturated benzoic acid (Sigma) and used for preparation of the calibration curve. Samples (200 µl) were mixed with 5 ml color reagent and subsequently heated at 100° for 4 min. The reaction was rapidly cooled to room temperature in iced water, and the absorbance at 540 nm was recorded.

### Xylose chemical genomic and *XYLA* and *XYLA,XKS1* synthetic genetic interaction screens

Robotic manipulation of the deletion mutant array was conducted using a Singer RoToR HDA (Singer Instruments). For the genome-wide *XYLA* and *XYLA,XKS1* SGA screens, the *MAT***a** query strain Y7092 (Tong and Boone 2006) was transformed with either *pXYLA* (YKB2179) or *pXYLA,XKS1* (YKB2178). The resulting query strains were mated to the *MAT***a** deletion mutant array, and SGA methodology (Tong *et al.* 2001; Tong *et al.* 2004) was used with the modifications previously described to maintain selection of the plasmid (Measday *et al.* 2005). After the final round of pinning on SD-uracil (2% glucose), the DMA containing the plasmids were subsequently pinned onto plates containing 2% xylose (SX-uracil). To identify deletion mutants that displayed growth defects or advantages on xylose, the deletion mutant array was also screened directly on 2% xylose plates. All three genome-wide screens were performed in triplicate at 30°, and growth was visually scored for slow growth, lethality, or suppression after one day on glucose and two days on xylose. Putative genetic interactions identified in a minimum of two out of three replicates in any of the five screens (DMA on xylose, p*XYLA* on glucose, *pXYLA,XKS1* on glucose, *pXYLA* on xylose, and *pXYLA,XKS1* on xylose) were confirmed in all five conditions. In brief, the deletion mutant was transformed using traditional methods (Gietz and Schiestl 2007) with the vector control pRS415 (Sikorski and Hieter 1989), *pXYLA*, or *pXYLA,XKS1*, and a series of dot assays were performed on SC-uracil media with either 2% glucose or 2% xylose as the sole carbon source at 30°. Confirmed genetic interactions and xylose sensitivity are listed insupporting information,Table S1.

### Aerobic xylose fermentation

Independent yeast colonies, isolated from YPD-agar plates (1% yeast extract, 2% peptone, 2% glucose, 2% agar), were first cultivated in 50 ml conical tubes containing 15 ml YEP (1% yeast extract, 2% peptone) medium supplemented with 2% (20 g/l) glucose at 30°, 200 rpm. Inoculum cultures were started by transferring 500 µl of the tube-grown cultures into 250 ml flasks containing 25 ml YEP medium supplemented with 2% (20 g/l) xylose or 2% (20 g/l) glucose (depending on the sugar to be used in the batch fermentations) and incubated for 24 hr at 30° and 200 rpm. The cells from these precultures were harvested by centrifugation at 18,000 *g* for 5 min at 4°, washed twice with sterile YEP media, and then used to inoculate final batch fermentations at an initial OD_620nm_ = 0.1. Batch fermentations were performed using 250 ml flasks containing 50 ml of YEP medium supplemented with 2% (20 g/l) of the desired carbon source (xylose or glucose) as indicated and incubated in a rotary shaker at 30° and 200 rpm. Samples for determination of biomass and metabolite concentration were periodically withdrawn under sterile conditions. The batches were terminated when no further changes in OD were observed, as after the sugar has been depleted the cells can begin to consume the ethanol. The data plotted were observed by reading until the maximum concentration of ethanol was observed.

### Kinetic parameters calculation

For the characterization of the strains used in this work, specific rates of growth (µ), glucose consumption (*q*_Glc_), xylose consumption (*q*_Xyl_), ethanol production (*q*_EtOH_), yield of ethanol on glucose (Y_EtOH/Glc_), and yield of ethanol on xylose (Y_EtOH/Xyl_) were determined. The µ, *q*_Glc_, and *q*_Xyl_ values were calculated during exponential growth phase. Because growth rates and ethanol production kinetics differed among studied strains, *q*_EtOH_, Y_EtOH/Glc_, and Y_EtOH/Xyl_ were calculated considering only the ethanol production phase, defined as the period from starting one sample before ethanol was detected up to the point when a sharp decrease in ethanol accumulation was observed. Following the same criteria, plots were constructed using only the data corresponding to the ethanol production phase. Flask cultures were performed at least in duplicate. The values reported represent the means of the experiments performed.

### Biomass and metabolite analyses

Cell growth was followed as optical density at 620 nm (spectrophotometer GENESYS20, ThermoFisher Scientific). Biomass was determined as dry-cell weight as described previously (Parachin *et al.* 2010). Glucose, xylose, ethanol, xylitol, acetate, and glycerol were analyzed by high-performance liquid chromatography (HPLC) (UltiMate 3000, Dionex) with refractive index detector (Shodex). Samples were loaded onto an Aminex HPX-87H ion exchange column (BioRad) operated at 42° and eluted with 5 mm H_2_SO_4_ at a flow rate of 0.4 ml/min.

## Results

### Genome-wide chemical and synthetic interaction analysis identifies novel regulators of xylose utilization

Our goal was to use SGA methodology to systematically screen the laboratory S288C yeast deletion mutant array to identify genetic determinants of xylose fermentation. As S288C has limited ability to utilize xylose (Chiang *et al.* 1981; Gong *et al.* 1983; Wang *et al.* 1980), our first goal was to construct a SGA query strain (Tong *et al.* 2001) with improved growth on xylose. Two plasmids, one expressing the *Piromyces sp*. E2 XI gene *XYLA* and the other expressing the combination of *XYLA* and the endogenous XK gene *XKS1*, were introduced into the wild-type laboratory strain (A, B inFigure S1). A series of growth curve analyses determined that, although the addition of p*XYLA* endowed growth on xylose, those cells expressing both *XYLA* and additional *XKS1* (p*XYLA,XKS1*) displayed a further improvement in growth on xylose (Figure S2). Similarly, in aerobic batch fermentations, strains containing p*XYLA* or p*XYLA,XKS1* displayed 14-fold and 15-fold improvement in xylose consumption, respectively, compared to the wild-type strains (C inFigure S2). These results demonstrate that the introduction of *XYLA* and additional *XKS1* improves the ability of the SGA query strain to utilize xylose as a carbon source, and they replicate previous studies using exogenous XI genes (reviewed in Van Maris *et al.* 2007).

**Figure 2:**
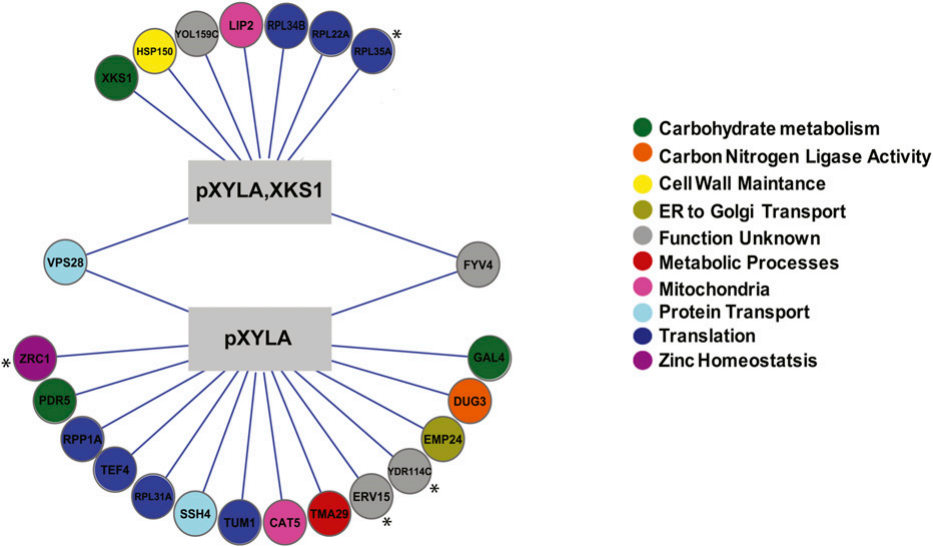
Genetic interaction network of p*XYLA* and p*XYLA,XKS1* on glucose. Genome-wide synthetic interaction SGA screens were performed using query strains that were transformed with p*XYLA* (YKB2179) and p*XYLA,XKS1* (YKB2178). Genes are represented by nodes that are color-coded according to their SGD cellular roles (www.yeastgenome.org) and/or assigned through review of the literature. Interactions are represented by edges. Deletion mutants that display synthetic lethal interactions are indicated by *.

**Figure 3:**
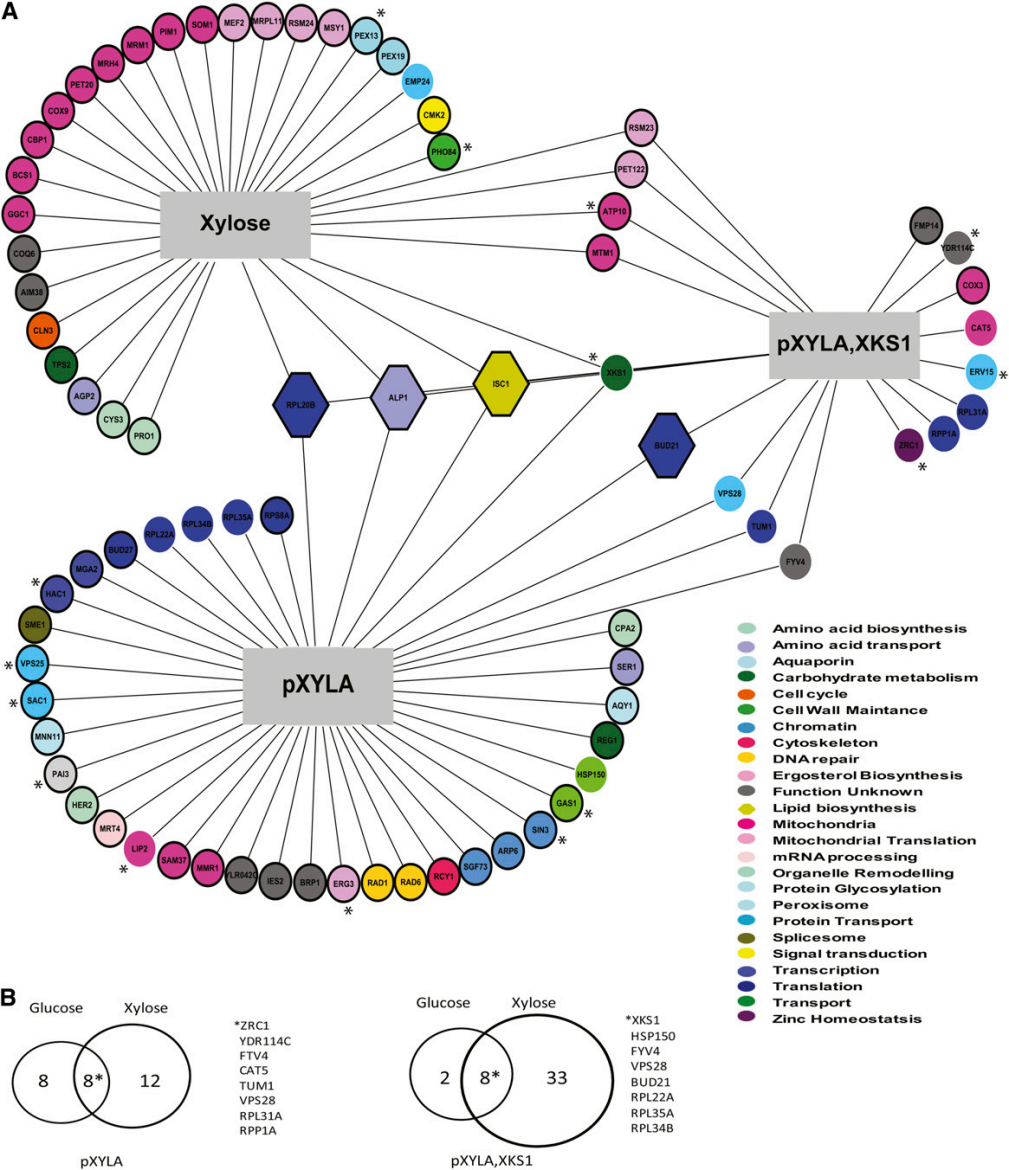
Xylose sensitivity and genetic interaction network of p*XYLA* and *pXYLA,XKS1* on xylose. (A) Genome-wide synthetic interaction SGA screens were performed using query strains that were transformed with p*XYLA* (YKB2179) and p*XYLA,XKS1* (YKB2178) and screened xylose plates. In addition, a chemical genomic screen was performed screening the deletion mutant array directly on xylose plates. Genes are represented by nodes that are color-coded according to their SGD cellular roles (www.yeastgenome.org) and/or assigned through review of the literature. Interactions are represented by edges. For deletion mutants displaying SG or SL interactions, the nodes are circles. For the deletion mutants that display improved growth phenotype (suppressors), the nodes are hexagons. Genes that interact with *pXYLA* or *pXYLA,XKS1* only on xylose have their nodes outlined in black. Deletion mutants that display synthetic lethal interactions are indicated by *. (B) Venn diagrams showing the number of overlapping genes in each of the screens performed: p*XYLA* on xylose and glucose and p*XYLA,XKS1* on xylose and glucose.

We performed genome-wide SL-SGA screens with both p*XYLA*- and p*XYLA,XKS1*-containing query strains. In brief, query strains containing p*XYLA* or p*XYLA,XKS1* were mated to the yeast deletion mutant array, and the SGA methodology was used to incorporate the plasmids into the deletion mutants (Tong *et al.* 2001). The growth of the deletion mutants containing the plasmids was tested on both glucose (Figure 2) and xylose (Figure 3) media. As a control, the deletion mutant array (without p*XYLA* or p*XYLA,XKS1*) was also screened for growth on xylose. Deletion mutants or deletion mutant–plasmid combinations that resulted in synthetic lethality (SL), slow growth (SG), or improved growth (suppressor) were identified (see *Materials and Methods*). To confirm the chemical and genetic interactions, regardless of which screen the mutant was identified in, each mutant was independently transformed with three different plasmids (vector control, p*XYLA*, and *pXYLA,XKS1*), dot assays were performed on plates containing either 2% glucose or 2% xylose as the carbon source, and growth was scored (seeTable S1 for a full list of interactions).

The addition of *pXYLA* or *pXYLA,XKS1* negatively affected the growth of 23 deletion mutants on glucose, and each plasmid had largely distinct genetic interactions (Figure 2). The addition of *pXYLA* resulted in slow growth in 13 deletion mutants and was lethal to deletion mutants of the vacuole zinc transporter *ZRC1* (MacDiarmid *et al.* 2000) and the uncharacterized genes *ERV15* and *YDR114c*. While additional *XKS1* relieved the lethality in these three strains, it caused lethality in deletion mutants of the large 60S ribosomal subunit *RPL35A* (Venema and Tollervey 1999) and slow growth in 8 additional deletion mutants. Only deletion mutants of the ESCRT-I component *VPS28* and the uncharacterized gene *FYV4* were sensitive to both plasmids. This result suggests that, although the addition of the exogenous gene *XYLA* and increased *XKS1* expression did not detectably impact the growth rate or consumption of glucose in a S288C wild-type background (Figure S2), their addition is not neutral and has cellular consequences in certain deletion mutant backgrounds. Although there have been contradictory reports regarding the impact of *XKS1* overexpression on growth rate and xylose consumption (Ho *et al.* 1998; Johansson *et al.* 2001; Matsushika and Sawayama 2008; Richard *et al.* 2000; Rodriguez-Pena *et al.* 1998; Toivari *et al.* 2001), it is clear that *XKS1* driven by the *TPI1* promoter not only increases xylose consumption (C inFigure S2) but also results in a synthetic dosage effect in many mutant backgrounds.

The majority of strains in the deletion mutant array could grow, albeit slowly, on media where the sole carbon was 2% xylose. We found that 4 deletion mutants were not viable (*ATP10*, *PEX13*, *PHO84*, and *XKS1*) on xylose and 26 deletion mutants displayed slow growth on xylose (Figure 3,Table S1). Remarkably, the vast majority of xylose-sensitive mutants could be rescued by the addition of *pXYLA* or *pXYLA,XKS1* plasmid. The exception to this was *XKS1*. As expected, *xks1Δ* cells could not grow on 2% xylose, but they could be partially rescued by p*XYLA* or *pXYLA,XKS1*. As *pXYLA,XKS1* could only partially rescue *xks1Δ* on xylose, it suggests that both copies of *XKS1*, the endogenous genomic copy and the plasmid-borne copy, are necessary to promote maximum xylose utilization S288C.

Similar to the results on glucose, *pXYLA* and *pXYLA,XKS1* displayed largely distinct genetic interactions on xylose (Figure 3A). While p*XYLA* negatively affected the growth of 16 deletion mutants on xylose (3 SL and 13 SG), *pXYLA,XKS1* negatively affected the growth of 37 deletion mutants on xylose (8 SL and 29 SG). For both plasmids, the genetic interactions they displayed on glucose were not all replicated on xylose media (Figure 3B). This result indicates that the detrimental effects of *XYLA* or *XKS1* expression on glucose in certain deletion mutant backgrounds can be alleviated by growth on xylose. The p*XYLA* and *pXYLA,XKS1* xylose interaction network is not limited to known players in carbohydrate metabolism; rather, the network implies that genes associated with diverse cellular pathways are required for XI-mediated xylose metabolism, including translation, DNA repair, and protein transport. For example, of the 53 genes identified between the two plasmids, FUNSPEC analysis (Robinson *et al.* 2002) indicates that a significant number encode components of the cytoplasmic ribosomal large subunit (*P* = 4.1e^−5^) or are implicated in the GO biological process of translation elongation (*P* = 0.003805). This chemical and functional genomics study dramatically expands our knowledge of proteins and pathways that are required for xylose utilization and optimal function of the XI/XK pathway.

### Xylose utilization is inhibited by suppressors in S288C

**Figure 4:**
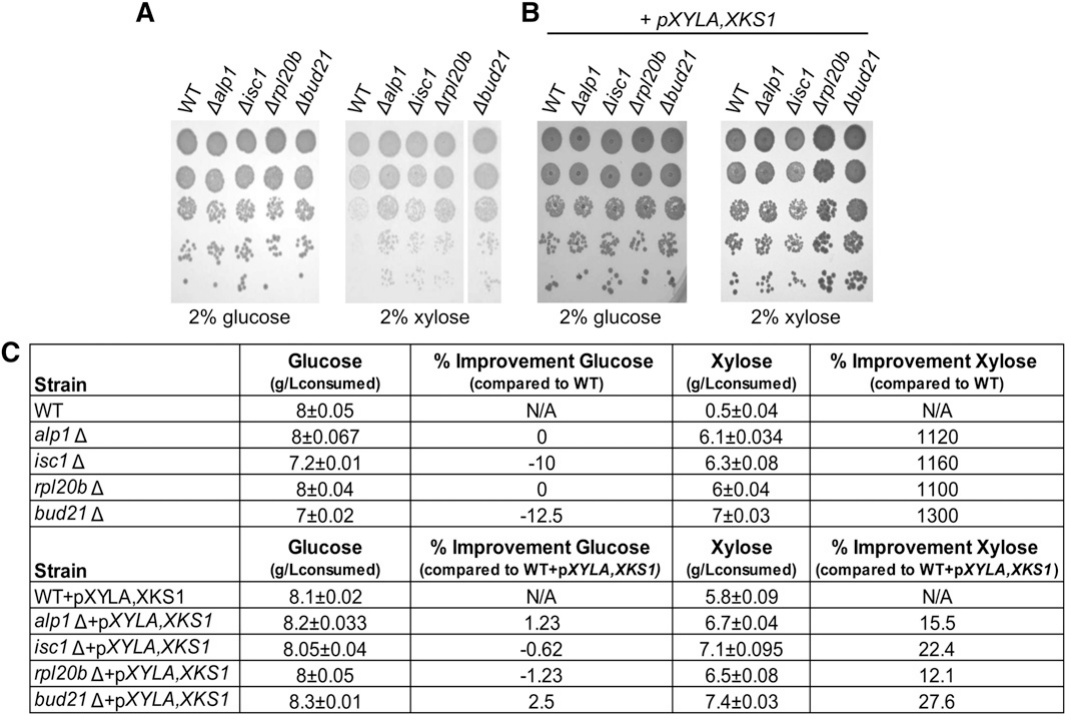
Deletion of *ALP1*, *BUD21*, *ISC1*, or *RPL20B* improve the xylose utilization of S288C yeast even in the absence of exogenous XI. Wild-type (WT, Y7092), *alp1*Δ (YKB2069), *isc1*Δ (YKB2530), *rpl20b*Δ (YKB 2531), and *bud21*Δ (YKB2532) cells without (A) or with *pXYLA,XKS1* (B) were plated in 10-fold serial dilutions onto YEP plates containing glucose or xylose. Plates were incubated at 30° for 1 day on glucose and 2 days on xylose. (C) Analysis of the consumption of glucose and xylose of the four suppressor mutants, *alp1*Δ (YKB2069), *isc1*Δ (YKB2530), *rpl20b*Δ (YKB 2531), and *bud21*Δ (YKB2532), compared to wild-type (WT, Y7092), either alone or transformed with *pXYLA,XKS1*. Growth cultures were started in either 13% glucose or xylose media, pitched with 1.5 × 10^7^ cells/ml and carried out at 30° with for 168 hr. The calculation for g/l of sugars consumed corresponds to the concentrations of sugars observed at the end of the batches compared to the starting amount.

The genome-wide chemical and genetic screens identified four deletion mutants that improved growth on xylose: *RPL20B*, *ALP1*, *ISC1*, and *BUD21*. All four putative suppressors, which to date have not been linked to pentose metabolism, are implicated in three diverse biological processes.Alp1 is an arginine transporter (Regenberg *et al.* 1999),Isc1 is a mitochondrial membrane–localized inositol phosphosphingolipid phospholipase C (Betz *et al.* 2002),Rpl20B is a component of the large (60S) ribosomal subunit (Lecompte *et al.* 2002; Planta and Mager 1998; Venema and Tollervey 1999), andBud21 is a component of the small ribosomal subunit (SSU) processosome (Dragon *et al.* 2002). To confirm that the null mutants of these genes confer advantageous growth on xylose, the genes were directly knocked out of a S288C laboratory strain, and a series of growth analyses and sugar consumption assays were performed (Figure 4). Deletion of these genes did not detectably impact growth on glucose (Figure 4A), and only deletion of *ISC1* and *BUD21* resulted in minor decreases in glucose consumption (Figure 4C). However, when the suppressor strains were grown on xylose media, they grew better than the wild-type strain (Figure 4A) and had dramatic improvements in xylose consumption (Figure 4C). We next asked if these suppressors could improve the consumption of xylose over and above that conferred by p*XYLA,XKS1*. Though the suppressors in combination with *pXYLA,XKS1* did not dramatically improve growth on xylose, the deletion of *BUD21* and *RPL20B* displayed reproducibly minor improvements of growth on xylose (Figure 4B). The suppressors of all these genes improved the consumption of xylose of strains transformed with *pXYLA,XKS1* from a modest 12% for *RPL20B* to 27.5% for *BUD21*. We next asked if a combination of the suppressors might result in a further improvement of xylose utilization. Though no double mutant was deleterious to growth on glucose or xylose media (data not shown), improvements in xylose consumption were either negligible or modest at best (Table S2). This finding suggests that the proteins encoded by these genes, although not presently linked, may be working through the same pathway or mechanisms to inhibit xylose consumption in S288C *S. cerevisiae*.

### Bud21 suppresses xylose utilization and fermentation in CEN.PK 113-3D

**Figure 5:**
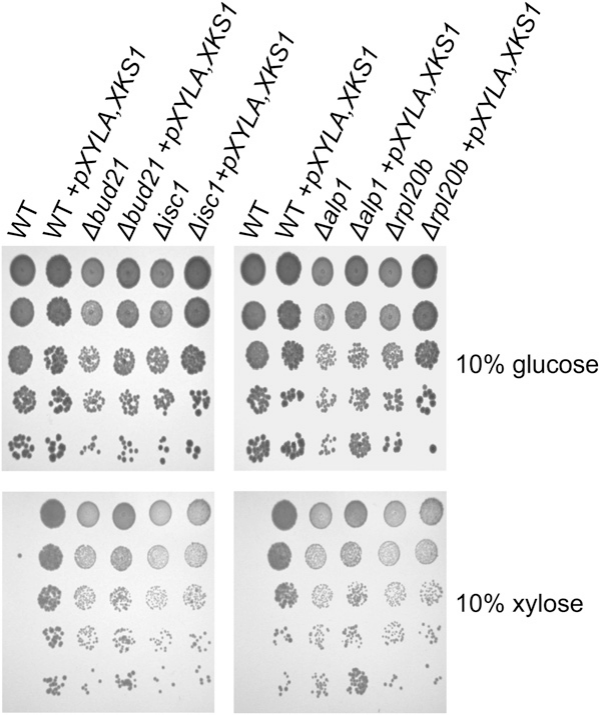
Deletion of *ALP1*, *BUD21*, *ISC1*, or *RPL20B* improve the xylose utilization of CEN.PK yeast. CEN.PK 133-13D wild-type (WT, YKB2684), *alp1Δ* (YKB2668), *isc1Δ* (YKB2670), *rpl20bΔ* (YKB2665), and *bud21Δ* (YKB2666) cells, alone or transformed with *pXYLA,XKS1*, were plated in 10-fold serial dilutions onto YEP plates containing glucose or xylose. Plates were incubated at 30° for 1 day on glucose and 2 days on xylose.

We next wanted to determine whether the suppressors identified in S288C also functioned as suppressors of xylose utilization in other yeast strains. For this purpose, we utilized the ethanol-tolerant *MAT***a** CEN.PK 113-3D strain (Van Dijken *et al.* 2000) that has previously been used in numerous xylose fermentation studies (Eliasson *et al.* 2000; Johansson *et al.* 2001; Van Vleet *et al.* 2008). Deletion mutants in CEN.PK 133-3D (hereafter called CEN.PK) were generated for *BUD21*, *ISC1*, *ALP1*, and *RPL20b* and subsequently transformed with *pXYLA,XKS1*. As seen for S288C, deletion of the suppressors resulted in the dramatic improvement of the ability of CEN.PK to grow on xylose plates, even in the absence of *pXYLA,XKS1* (Figure 5).

**Figure 6:**
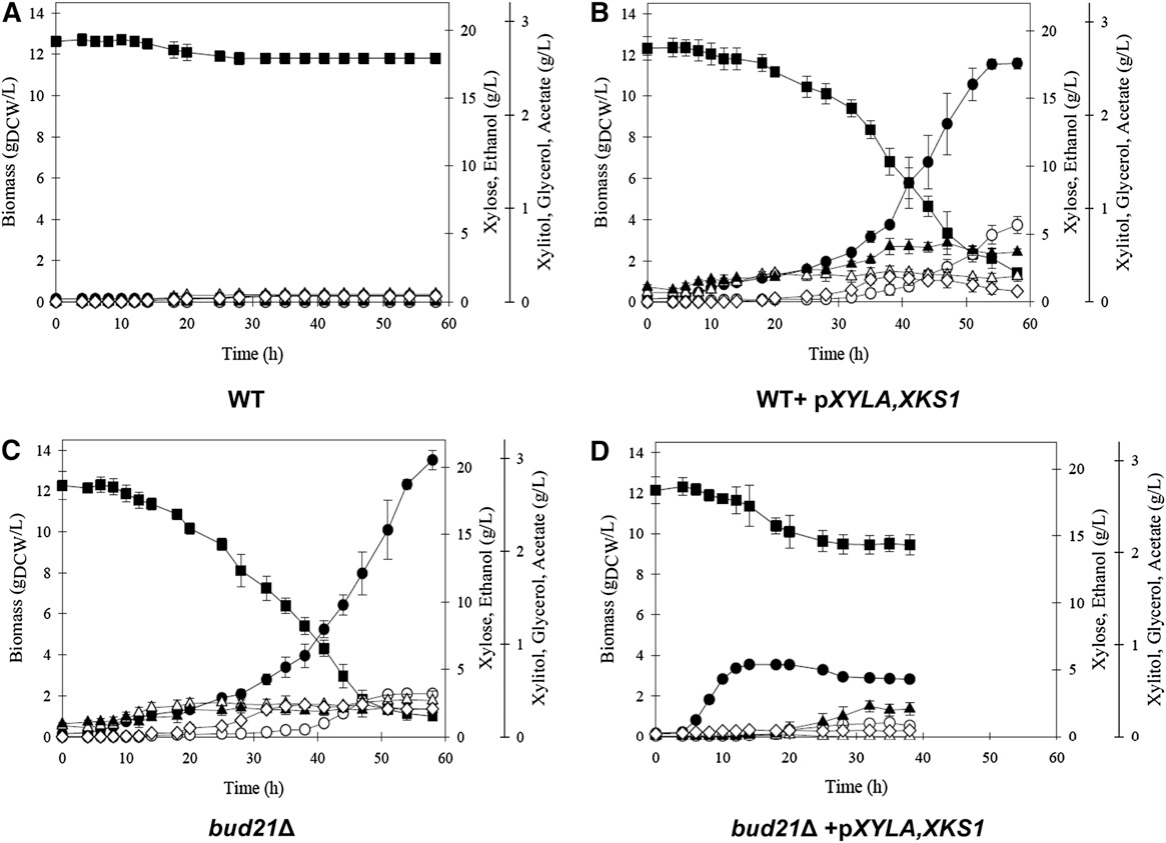
Deletion of *BUD21* improves the xylose fermentation of CEN.PK yeast. Fermentation profile of the *S. cerevisiae* CEN.PK 113-13D derivative strains during aerobic batch cultivation in xylose (20 g/l). (A) CEN.PK 113-13D wild-type (WT, YKB2684), (B) WT transformed with *pXYLA,XKS1* (YKB2680), (C) *bud21Δ* (YKB2666), and (D) *bud21Δ* transformed with *pXYLA,XKS1* (YKB2667). Biomass (●), xylose (▪), ethanol (○), glycerol (△), acetate (▴), and xylitol (⋄). The batches were terminated when no further changes in OD were observed. Each data point represents the mean ± SD from triplicate experiments.

We next performed a series of controlled aerobic batch fermentation studies to assess whether one of the suppressors could improve xylose fermentation. *BUD21* was selected as its deletion resulted in the greatest improvement in xylose consumption in the S288C background with and without the addition *pXYLA,XKS1* (Figure 4). The fermentations were performed using CEN.PK and CEN.PK *bud21Δ* with and without *pXYLA,XKS1* in both glucose (Table 2,Figure S3) and xylose (Table 3, Figure 6). For xylose and glucose fermentation, the concentrations of xylose or glucose, ethanol, acetate, and glycerol were measured, along with the cell biomass. In addition, xylitol levels were measured in the xylose fermentation. During glucose fermentation, the addition of *pXYLA,XKS1* or deletion of *BUD21* resulted in an increment in the specific growth rate and final biomass levels compared with the control strain; however, this rise in biomass production resulted in nearly identical decreases in both ethanol and glycerol production (Table 2,Figure S3). Surprisingly, the CEN.PK strain containing both the *bud21Δ* and *pXYLA,XKS1* not only caused a further reduction in ethanol and glycerol production but also a dramatic increase in acetate production. This increase may reflect the increase in growth rate of this strain and the associated energetic demand.

**Table 2:** Kinetic parameters from *Saccharomyces cerevisiae* CEN.PK 113-13D derivative strains grown aerobically in YEP medium supplemented with glucose 20 g/l

Strain	Biomass*^a^* (g_DCW_/l)	µ (h^−1^)	*q*_Glc_ (g_Glc_/g_DCW_⋅h)	*q*_EtOH_ (g_EtOH_/g_DCW_−h)	Acetate*^a^* (g/l)	Glycerol*^a^* (g/l)	Ethanol*^a^* (g/l)	Y_EtOH/Glc_ (g_EtOH_/g_Glc_)
*WT*	5.018 ± 0.184	0.354 ± 0.041	0.236 ± 0.019	0.144 ± 0.018	0.495 ± 0.022	1.429 ± 0.313	10.586 ± 1.683	0.535 ± 0.071
*WT pXYLA,XKS1*	6.791 ± 0.345	0.420 ± 0.004	0.238 ± 0.020	0.128 ± 0.002	0.732 ± 0.032	0.637 ± 0.016	7.609 ± 0.121	0.443 ± 0.011
*bud21Δ*	6.915 ± 0.170	0.416 ± 0.002	0.329 ± 0.005	0.134 ± 0.006	0.670 ± 0.055	0.705 ± 0.080	7.647 ± 0.121	0.473 ± 0.031
*bud21Δ pXYLA,XKS1*	7.978 ± 0.046	0.549 ± 0.009	0.131 ± 0.003	0.052 ± 0.003	2.810 ± 0.120	0.201 ± 0.055	5.809 ± 0.308	0.326 ± 0.001

Aerobic batch fermentations were performed in YEP supplemented with 2% glucose as described in the *Materials and Methods* using CEN.PK 133-13D wild-type (WT, YKB2684), WT transformed with *pXYLA,XKS1* (YKB2680), *bud21Δ* (YKB2666), and *bud21Δ* transformed with *pXYLA,XKS1* (YKB2667). Values listed are the average ± SE of duplicate experiments. Specific rates of growth (µ), glucose consumption (*q*_Glc_), ethanol production (*q*_EtOH_), and yield of ethanol on glucose (Y_EtOH/Glc_) were determined. Values of µ and *q*_Glc_ were calculated during exponential growth phase.

a Values obtained at the end of each fermentation.

**Table 3:** Kinetic parameters from *Saccharomyces cerevisiae* CEN.PK 113-13D derivative strains grown aerobically in YEP medium supplemented with xylose 20 g/l

Strain	Biomass*^a^* (g_DCW_/l)	µ (h^−1^)	*q*_Xyl_ (g_Xyl_/g_DCW_⋅h)	*q*_EtOH_ (g_EtOH_/g_DCW_−h)	Xylitol*^a^* (g/l)	Acetate*^a^* (g/l)	Glycerol*^a^* (g/l)	Ethanol*^a^* (g/l)	Y_EtOH/Xyl_ (g_EtOH_/g_Xyl_)
*WT*	0.298 ± 0.014	0.018 ± 0.02	ND	ND	0.000 ± 0.000	0.000 ± 0.000	0.000 ± 0.000	0.000 ± 0.000	ND
*WT pXYLA,XKS1*	11.567 ± 0.199	0.132 ± 0.006	0.031 ± 0.001	0.010 ± 0.000	0.580 ± 0.164	0.525 ± 0.038	0.264 ± 0.048	5.295 ± 0.425	0.352 ± 0.003
*bud2Δ1*	12.922 ± 0.117	0.143 ± 0.006	0.031 ± 0.001	0.006 ± 0.001	1.350 ± 0.083	0.301 ± 0.042	0.399 ± 0.070	3.189 ± 0.170	0.201 ± 0.017
*bud21Δ pXYLA,XKS1*	2.841 ± 0.04	0.265 ± 0.004	0.061 ± 0.005	0.017 ± 0.004	0.294 ± 0.048	0.297 ± 0.019	0.000 ± 0.000	1.033 ± 0.041	0.315 ± 0.018

Aerobic batch fermentations were performed in YEP supplemented with 2% xylose as described in *Materials and Methods* using CEN.PK 113-13D wild-type (WT, YKB2684), WT transformed with *pXYLA,XKS1* (YKB2680), *bud21Δ* (YKB2666), and *bud21Δ* transformed with *pXYLA,XKS1* (YKB2667). Values are the average ± SE of triplicate experiments. Specific rates of growth (µ), xylose consumption (*q*_Xyl_), ethanol production (*q*_EtOH_), and yield of ethanol on xylose (Y_EtOH/Xyl_) were determined. Values of µ and *q*_Xyl_ were calculated during exponential growth phase.

a Values obtained at the end of each fermentation.

As expected, the control CEN.PK strain showed no increase in biomass or ethanol production in xylose (Figure 6, Table 3). In case of the CEN.PK strain containing p*XYLA,XKS1*, there was a significant increase in biomass and ethanol production (5.295 g/l), and a low level of xylitol was detected in the cells at the end of the fermentation (Figure 6, Table 3). The greatest biomass increase was observed for the suppressor-deficient strain *bud21Δ*, once again confirming that the strain is capable of sustained growth in xylose media. Although the *bud21Δ* strain was also able to produce ethanol (3 g/l), its ethanol production may be hindered due to a buildup of xylitol in the cells (Table 3), which may in turn be the result of a redox imbalance in the cells. Unfortunately, the combination of *bud21Δ* and p*XYLA,XKS1* did not result in an improvement in fermentative capacity in xylose (Figure 6D). Despite the fast initial burst in growth (increased μ), biomass did not increase over the course of the experiment and ethanol production was low (1 g/l), although its overall ethanol yield (Y_EtOH_/_Xyl_) was nearly equivalent to the CEN.PK *pXYLA,XKS1* strain (Table 3). Although combining the XI with the deletion of the suppressor *BUD21* did not lead to additive ethanol production, the SGA screen identified a deletion mutant that can consume xylose and produce ethanol at production levels and kinetics similar to strains containing exogenous xylose pathway genes (Matsushika *et al.* 2009). Not only does this result justify the continued use of functional genomics as a tool for identifying novel genetic strategies to improve industrial yeast strains but it also implies that *S. cerevisiae* may not require the addition of exogenous genes for efficient xylose fermentation.

## Discussion

The majority of studies to date on improving xylose utilization in *S. cerevisiae* have taken a candidate gene or directed approach (reviewed in Van Vleet and Jeffries 2009). Unfortunately, these approaches are limited by our knowledge of the pathways/proteins regulating xylose utilization. To our knowledge, this is the first study to apply genome-wide chemical and synthetic genetic interaction screens to systematically assess the contribution of nonessential genes to xylose utilization via both endogenous and exogenous XI pathways. Indeed, despite the wealth of genetic interactions generated by large-scale SGA screening (Costanzo *et al.* 2010; Tong *et al.* 2001; Tong *et al.* 2004), only four genetic interactions have been identified for *XKS1* (Costanzo *et al.* 2010; Fiedler *et al.* 2009), which may reflect the fact that most SGA screens have been conducted on standard glucose media.

We found that, in spite of the numerous reports indicating that laboratory *S. cerevisiae* strains cannot utilize xylose (Batt *et al.* 1986; Chiang *et al.* 1981; Gong *et al.* 1983; Wang *et al.* 1980), the vast majority of deletion mutant strains could survive when xylose was the sole carbon source (Figure 3A). This is not unexpected as the *S. cerevisiae* genome encodes a putative xylose utilization pathway (Figure 1) and many members of the *Saccharomyces sensu stricto* group have the ability to utilize xylose as a carbon source (Attfield and Bell 2006; Wenger *et al.* 2010). While *XKS1* was identified as being essential for growth on xylose, no other genes encoding components of endogenous xylose pathway (Figure 1) were identified in any of the screens. This is likely because there are multiple putative redundant genes that encode for xylose reductase and D-xylitol dehydrogenase, whereasXks1 may be the sole xylulokinase. That said, *pXYLA* alone could rescue *xks1Δ* growth on xylose, which suggests that there may be alternative proteins/pathways in yeast that can convert D-xylulose and that there may be alternative noncanonical XR and XDH genes, too. Many of the xylose-sensitive deletion mutants are, as expected, implicated in mitochondrial functions because, during growth on xylose, the redox cofactors NADH and NADPH are involved in catabolism and biosynthesis (Mo *et al.* 2009; Salusjarvi *et al.* 2003; Shi *et al.* 2002). Remarkably, the vast majority of xylose-sensitive mutants could be rescued by the addition of *pXYLA* or *pXYLA,XKS1* plasmid. One interpretation of this result is that the proteins encoded by the xylose-sensitive genes are essential for or contribute to S288C’s endogenous xylose utilization pathway but that they do not detectably impact xylose utilization mediated when XI is present. Alternatively, as mitochondrial mutants display fitness defects on nonfermentable carbons sources (Steinmetz *et al.* 2002), the detected growth defect may not be specific to xylose but apply to any nonpreferred carbon source.

Using the SGA methodology, we systematically screened the contribution of nonessential yeast genes to XI/XK-mediated xylose metabolism (Figure 3A,Table S1). One striking feature of the network was the abundance of deletion mutants identified that encode proteins implicated in various aspects of translation. Similarly, genes implicated in translation were upregulated in *XDH1* xylose-positive strains (Wenger *et al.* 2010). This result suggests that translation plays a key role in remodeling the cell for xylose utilization, which is understandable in light of the dramatic changes in the transcriptome (Bengtsson *et al.* 2008; Jin *et al.* 2002; Jin *et al.* 2004; Kuyper *et al.* 2005; Runquist *et al.* 2009; Salusjarvi *et al.* 2006; Wenger *et al.* 2010) and proteome (Karhumaa *et al.* 2009; Salusjarvi *et al.* 2008; Salusjarvi *et al.* 2003) that occurred upon xylose exposure. This result may also explain the identification of genes implicated in chromatin remodeling or transcription and protein transport. Understanding why these proteins are required for *XYLA,XKS1*-dependent utilization on xylose may reveal novel genetic mechanisms to improve xylose utilization of industrial strains.

One of the most interesting findings of this study was the identification of four genes, *BUD21*, *ALP1*, *ISC1*, and *RPL20B*, which suppress the natural ability of yeast to utilize xylose. Deletion of these genes resulted in increased xylose consumption and growth on xylose media to levels similar to yeast containing the XI/XK system (Figures 4 and 5). Hence, there may be fitness disadvantages in retaining the ability to consume xylose that laboratory yeast has evolved to inhibit. How are the suppressors inhibiting xylose utilization? Of the four suppressors identified, *bud21Δ* had the greatest impact on xylose consumption (Figure 4).Bud21 is a component of the small ribosomal subunit (SSU) processosome that contains U3 snoRNA (Dragon *et al.* 2002), and expression of *BUD21* mRNA is upregulated in high-sucrose media, possibly due a strong osmotic response (Ando *et al.* 2006; Perez-Torrado *et al.* 2010).Bud21 is also involved in ribosomal protein biogenesis and processing, functions transiently repressed by stress conditions, such as those experienced during a fermentation environment (Hirasawa *et al.* 2010). Therefore, deletion of certain aspects of the stress response may be advantageous, allowing for bypassing some of the initial stress conditions that occur during xylose fermentation. Alternatively, both the XR/XDH and XI/XK xylose utilization pathways cause dramatic transcriptional remodeling of the cell (Bengtsson *et al.* 2008; Fendt and Sauer 2010; Hector *et al.* 2009; Jin *et al.* 2004; Pitkanen *et al.* 2003; Runquist *et al.* 2009; Salusjarvi *et al.* 2008; Salusjarvi *et al.* 2003; Wenger *et al.* 2010). Therefore, the suppressors may all be regulating some aspect of transcription or translation, which is likely the case ofRpl20b andBud21. Of course, these two possibilities are not mutually exclusive as the stress response is largely mediated by remodeling of the cells’ transcriptome and proteome. As robust stress-tolerance is essential for yeast to efficiently ferment cellulosic hydrolysates at an industrial scale, if the suppressors are functioning by modulating aspects of stress response, they may not be an appropriate genetic strategy for the improvement of industrial yeast strains. The mechanism by which these proteins inhibit xylose utilization in yeast will require further study to assess their value for the cellulosic biofuel industry.

We found that deletion of the suppressor *BUD21* improved growth on xylose and that CEN.PK *bud21Δ* cells were able to ferment xylose and produce ethanol (Figure 6, Table 3). Unfortunately, the combination of *bud21Δ* and *pXYLA,XKS1* did not result in further increases in ethanol production; rather, it was detrimental in the batch fermentations (Figure 6, Table 3). Though there was an initial fast burst in biomass accumulation, CEN.PK *bud21Δ pXYLA,XKS1* cells plateaued in growth much sooner than CEN.PK wild-type cells. This is surprisingly as neither S288C nor CEN.PK *bud21Δ* transformed with *pXYLA,XKS1* displayed growth defects on xylose plates (Figures 4 and 5). One possibility is that, although the cultures were aerobic, oxygen may be limiting at the high cellular concentrations of batch fermentations, effects that do not occur on plates. The exact reason for the differences in growth on plates and culture require further investigation. However, it is clear that, at least in the case of deletion of *BUD21*, activation of *S. cerevisiae*’s natural ability to utilize and ferment xylose is not synergistic with exogenous *pXYLA,XKS1* in aerobic xylose fermentations. It may be beneficial to exploit the molecular “barcodes” of yeast mutant collections and microarray-based methods (reviewed in Smith *et al.* 2010) to identify mutants that improve either recombinant XI/XK or XR/XDH/XK xylose fermentation in anaerobic batch cultures that better replicate industrial-scale biofuel production. Remarkably, the performance of the CEN.PK *bud21Δ* strain in batch fermentation in media in which xylose is the sole carbon source was similar or better than many recombinant yeast strains (Matsushika *et al.* 2009). This finding suggests the addition of exogenous genes may not be the sole method of improving the xylose fermentation efficiency of *Saccharomyces*; rather, efforts should focus on identifying and optimizing the endogenous xylose utilization pathway in yeast.

## Supplementary Material

Supporting Information
