# Supplementary material for: Chemical and Synthetic Genetic Array Analysis Identifies Genes that Suppress Xylose Utilization and Fermentation in Saccharomyces cerevisiae
Source: G3 (Bethesda). 2011 Sep 1;1(4):247–58. doi: 10.1534/g3.111.000695 (PMC3276145; doi:10.1534/g3.111.000695)
Supplement: Supporting Information [file supp_1_4_247__index.html]

Supporting Information 

# Chemical and Synthetic Genetic Array Analysis Identifies Genes that Suppress Xylose Utilization and Fermentation in *Saccharomyces cerevisiae*

## Supporting Information for Usher *et al.*, 2011

**Files in this Data Supplement:**

- Supporting Information - Figures S1-S3 and Tables S1 and S2 (PDF, 472 KB)
- Figure S1 - (A) Schematic map *pXYLA* (B) Schematic map of *pXYLA,XKS1* (PDF, 68 KB)
- Figure S2 - The addition of *pXYLA* or *pXYLA,XKS1* increases the xylose utilization of *S. cerevisiae* (PDF, 140 KB)
- Figure S3 - Fermentation profile of the *S. cerevisiae* CEN.PK 113-13D derivative strains during aerobic batch cultivation in glucose (20g/L) (PDF, 200 KB)
- Table S2 - Double mutants do not display additive improvement in xylose utilization (PDF, 48 KB)
- Table S1 - Confirmed genetic interactions (Microsoft Excel, .xls, 76 KB)
