# Supplementary material for: Chemical and Synthetic Genetic Array Analysis Identifies Genes that Suppress Xylose Utilization and Fermentation in Saccharomyces cerevisiae
Source: G3 (Bethesda). 2011 Sep 1;1(4):247–58. doi: 10.1534/g3.111.000695 (PMC3276145; doi:10.1534/g3.111.000695)
Supplement: Supporting Information [file supp_1.4.247_000695SI.pdf]

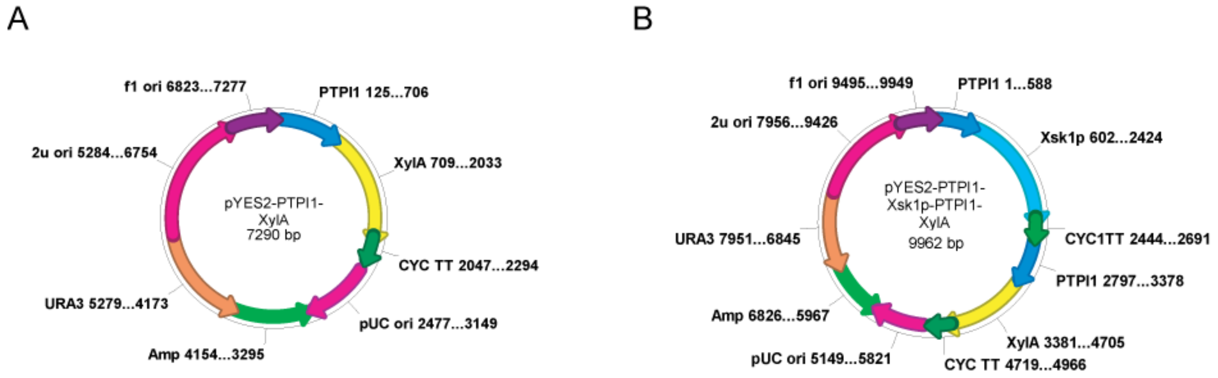


**Figure S1** **(A)** Schematic map *pXYLA*. The pYES2 plasmid containing the exogenous XI gene *XYLA* from *Piromyces SpE2.* **(B)** Schematic map of *pXYLA,XKS1*. The pYES2 plasmid containing the XI gene *XYLA* and an additional copy of the endogenous XK gene *XKS1* from *S. cerevisiae*.

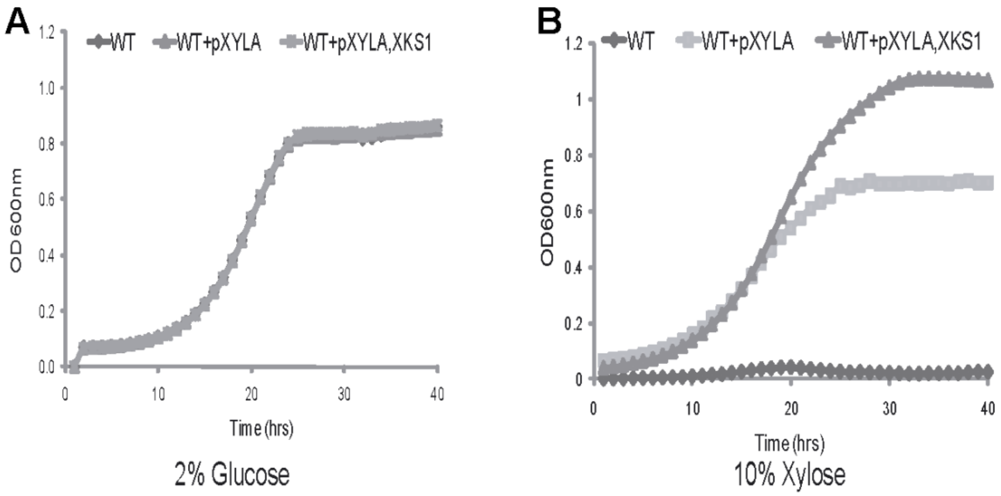


C

| Strain | Glucose (g/Lconsumed) | % Improvement Glucose (compared to WT) | Xylose (g/Lconsumed) | % Improvement Xylose (compared to WT) |
|---|---|---|---|---|
| WT | 9.5±0.05 | N/A | 0.6±0.09 | N/A |
| WT+p*XLYA* | 9±0.7 | -5.3 | 9±0.1 | 1400 |
| WT+p*XYLA,XKS1* | 9.7±0.1 | 2.1 | 9.9±0.02 | 1550 |

**Figure S2** The addition of p*XYLA* or p*XYLA,XKS1* increases the xylose utilization of *S. cerevisiae*. Growth analysis of wild-type (WT, Y7092), to transformed strains, WT + p*XYLA* (YKB2179) and WT + p*XYLA,XKS1* (YKB2178) in liquid media containing 2% glucose (**A**) or 10% xylose (**B**). Growth analysis was performed in triplicate on Multiskan Ascent plate reader (Thermo Electron Corporation), with $OD_{600}$ taken every 30 min over a 20-hour period at 30$^{o}$. The error bars represent 1 standard deviation and are smaller than the data marker. **(C)** Sugar consumption table of wild-type (WT, Y7092), or wild-type transformed with p*XYLA* (YKB2179), or p*XYLA,XKS1* (YKB2178) starting in either 13% glucose or xylose media. Fermentations were pitched with 1.5x$10^{7}$ cells/ml and carried out at 30°. The calculation for g/L consumed of sugars corresponds to the concentrations of sugars observed at the end of the batches compared to the starting amount.

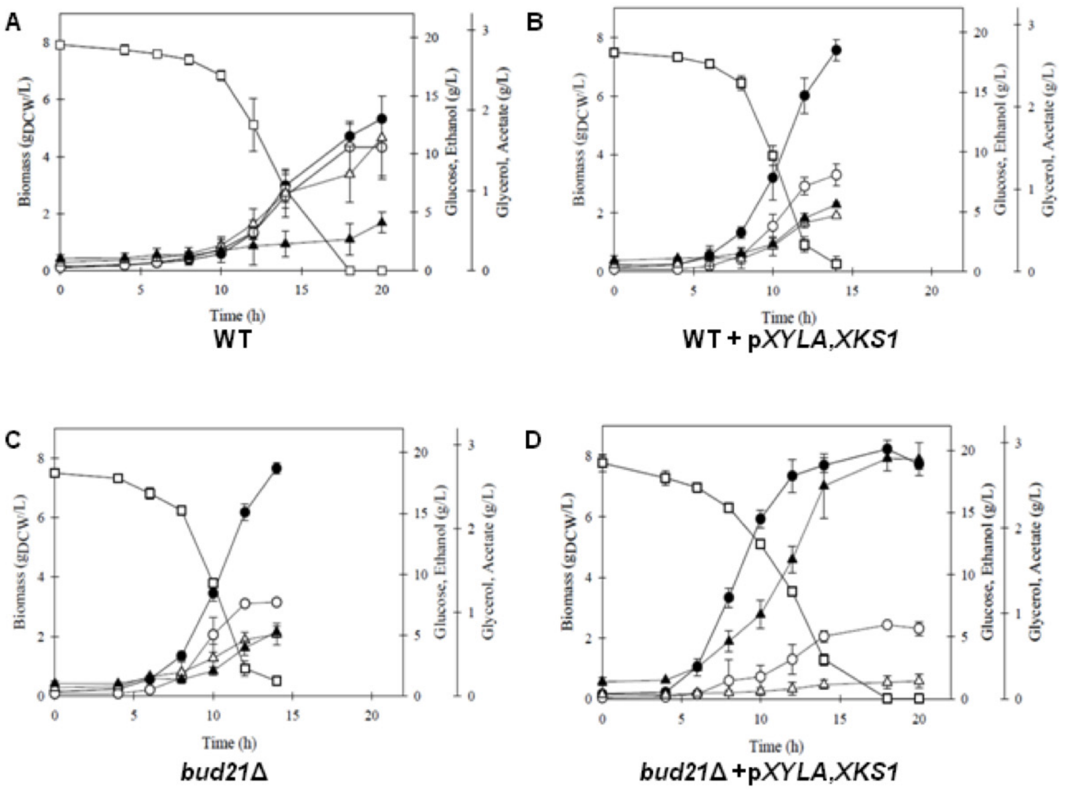


**Figure S3** Fermentation profile of the *S. cerevisiae* CEN.PK 113-13D derivative strains during aerobic batch cultivation in glucose (20g/L). **(A)** CEN.PK 113-13D wild-type (WT, YKB2684), (**B**) WT transformed with *pXYLA,XKS1* (YKB2680), (C) *bud21Δ* (YKB2666) and (D) *bud21Δ* transformed with *pXYLA,XKS1* (YKB2667). Biomass (*closed circles*), glucose (*open squares*), ethanol (*open circles*), glycerol (*open triangles*), and acetate (*closed triangles*). The batches were terminated when no further changes in OD was observed. Each data point represents the mean±SD from duplicated experiments.

**Table S1 Excel sheet containing the confirmed genetic interactions.**

Table S1 is available for download at http://www.g3journal.org/lookup/suppl/doi:10.1534/g3.111.000695/-/DC1/TableS1.xls.

Spreadsheets:

DMA-xylose = screen of the DMA on 2% xylose

XYLA - xylose = pXYLA SGA screen on 2% xylose

XYLA,XKS1 - xylose = pXYLA,XKS1 SGA screen on 2% xylose

XYLA - glucose = pXYLA SGA screen on 2% glucose

XYLA,XKS1 - glcuose = pXYLA,XKS1 SGA screen on 2% glucose

**Table S2 Double mutants do not display additive improvement in xylose utilization.**

| Strain | Glucose (g/L consumed) | Improvement Glucose (compared to WT) | Xylose (g/L consumed) | Improvement Xylose (compared to WT) |
|---|---|---|---|---|
| WT | 8±0.05 | N/A | 0.5±0.04 | N/A |
| *alp1Δ isc1Δ* | 7.1±0.015 | -11.25 | 6.5±0.05 | 1200 |
| *alp1Δ rpl20bΔ* | 7.2±0.02 | -10 | 6.7±0.08 | 1240 |
| *isc1Δ rpl20bΔ* | 7±0.06 | -12.5 | 6.6±0.074 | 1216 |
| *bud21Δ alp1Δ* | 8.1±0.09 | 1.25 | 6.2±0.06 | 1140 |
| *bud21Δ isc1Δ* | 8.4±0.08 | 5 | 7.15±0.15 | 1330 |
| *bud21Δ rpl20bΔ* | 8.3±0.1 | 3.75 | 6.5±0.06 | 1200 |

| Strain | Glucose (g/L consumed) | Improvement Glucose (compared to WT +p*XYLA,XKS1)* | Xylose (g/L consumed) | Improvement Xylose (compared to WT +p*XYLA,XKS1*) |
|---|---|---|---|---|
| WT +*pXYLA,XKS1* | 8.1±0.02 | N/A | 5.8±0.09 | N/A |
| *alp1Δ isc1Δ* +p*XYLA,XKS1* | 8.6±0.08 | 6.2 | 7.2±0.02 | 24.1 |
| *alp1Δ rpl20bΔ* +p*XYLA,XKS1* | 8.2±0.074 | 1.23 | 7±0.03 | 20.7 |
| *isc1Δ rpl20bΔ* +p*XYLA,XKS1* | 8.3±0.08 | 2.5 | 6.2±0.02 | 6.9 |
| *bud21Δ alp1Δ* +p*XYLA,XKS1* | 8.0±0.08 | -1.23 | 7.2±0.03 | 24.16 |
| *bud21Δ isc1Δ* +p*XYLA,XKS1* | 8.4±0.09 | 3.7 | 7.4±0.04 | 27.6 |
| *bud21Δ rpl20bΔ* +p*XYLA,XKS1* | 8.2±0.1 | 1.23 | 6.8±0.1 | 17.26 |

**Analysis of the consumption of glucose and xylose of the double mutants of the suppressors.** Strains tested were Wild-type (YKB731), *alp1Δ isc1Δ* (YKB2534), *alp1Δ rpl20bΔ* (YKB2535), *isc1Δ rpl20bΔ* (YKB2536), *bud21Δ alp1Δ* (YKB2537), *bud21Δ isc1Δ* (YKB2538), *bud21Δ rpl20bΔ* (YKB2539) without (top table) and with *pXYLA,XKS1* (bottom table). Growth cultures were started in either 13% glucose or xylsoe media, pitched with 1.5 x $10^7$ cells/ml and carried out at 30° with for 168 hours. The calculation for g/L consumed of sugars corresponds to the concentrations of sugars observed at the end of the batches compared to the starting amount.
