# Supplementary material for: Chemical and Synthetic Genetic Array Analysis Identifies Genes that Suppress Xylose Utilization and Fermentation in Saccharomyces cerevisiae
Source: G3 (Bethesda). 2011 Sep 1;1(4):247–58. doi: 10.1534/g3.111.000695 (PMC3276145; doi:10.1534/g3.111.000695)
Supplement: Supporting Information [file supp_1.4.247_FigureS1.pdf]

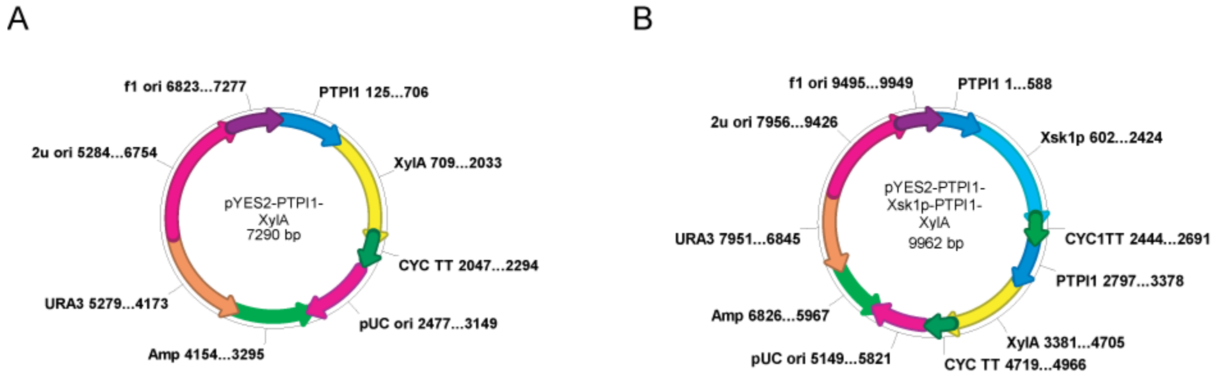

Figure S1 **(A)** Schematic map *pXYLA*. The pYES2 plasmid containing the exogenous XI gene *XYLA* from *Piromyces SpE2.* **(B)** Schematic map of *pXYLA,XKS1*. The pYES2 plasmid containing the XI gene *XYLA* and an additional copy of the endogenous XK gene *XKS1* from *S. cerevisiae*.
