# Supplementary material for: Chemical and Synthetic Genetic Array Analysis Identifies Genes that Suppress Xylose Utilization and Fermentation in Saccharomyces cerevisiae
Source: G3 (Bethesda). 2011 Sep 1;1(4):247–58. doi: 10.1534/g3.111.000695 (PMC3276145; doi:10.1534/g3.111.000695)
Supplement: Supporting Information [file supp_1.4.247_FigureS2.pdf]

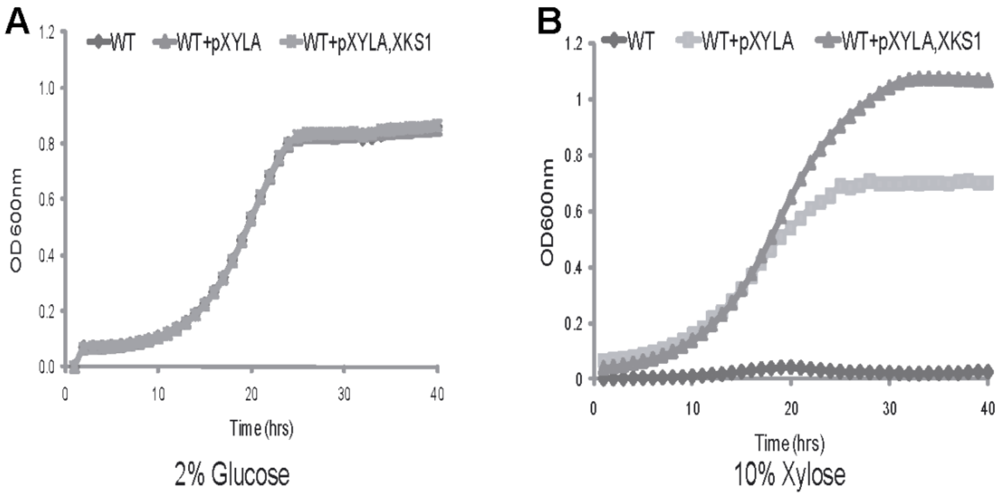

C

Strain	Glucose (g/Lconsumed)	% Improvement Glucose (compared to WT)	Xylose (g/Lconsumed)	% Improvement Xylose (compared to WT)
WT	9.5±0.05	N/A	0.6±0.09	N/A
WT+p*XLYA*	9±0.7	-5.3	9±0.1	1400
WT+p*XYLA,XKS1*	9.7±0.1	2.1	9.9±0.02	1550

Figure S2 The addition of p*XYLA* or p*XYLA,XKS1* increases the xylose utilization of *S. cerevisiae*. Growth analysis of wild-type (WT, Y7092), to transformed strains, WT + p*XYLA* (YKB2179) and WT + p*XYLA,XKS1* (YKB2178) in liquid media containing 2% glucose (**A**) or 10% xylose (**B**). Growth analysis was performed in triplicate on Multiskan Ascent plate reader (Thermo Electron Corporation), with OD_{600} taken every 30 min over a 20-hour period at 30°. The error bars represent 1 standard deviation and are smaller than the data marker. **(C)** Sugar consumption table of wild-type (WT, Y7092), or wild-type transformed with p*XYLA* (YKB2179), or p*XYLA,XKS1* (YKB2178) starting in either 13% glucose or xylose media. Fermentations were pitched with $1.5x10^7$ cells/ml and carried out at 30°. The calculation for g/L consumed of sugars corresponds to the concentrations of sugars observed at the end of the batches compared to the starting amount.
