# Supplementary material for: Chemical and Synthetic Genetic Array Analysis Identifies Genes that Suppress Xylose Utilization and Fermentation in Saccharomyces cerevisiae
Source: G3 (Bethesda). 2011 Sep 1;1(4):247–58. doi: 10.1534/g3.111.000695 (PMC3276145; doi:10.1534/g3.111.000695)
Supplement: Supporting Information [file supp_1.4.247_FigureS3.pdf]

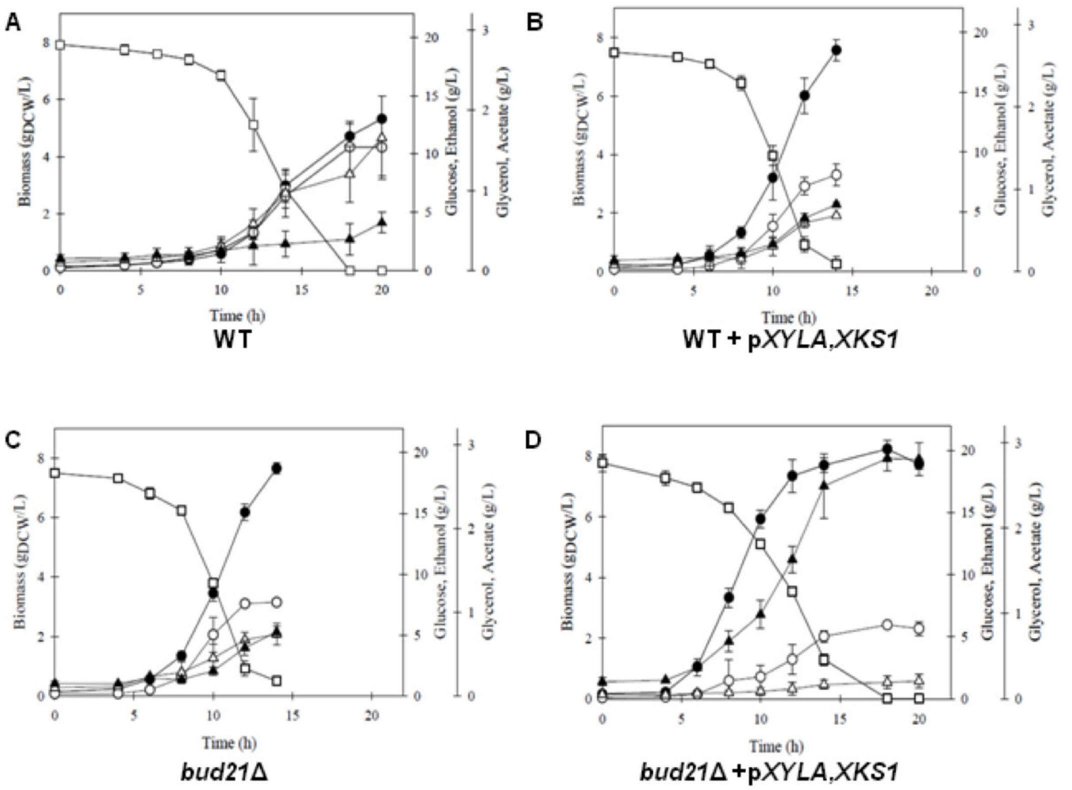

Figure S3 Fermentation profile of the *S. cerevisiae* CEN.PK 113-13D derivative strains during aerobic batch cultivation in glucose (20g/L). **(A)** CEN.PK 113-13D wild-type (WT, YKB2684), (**B**) WT transformed with *pXYLA,XKS1* (YKB2680), (C) *bud21Δ* (YKB2666) and (D) *bud21Δ* transformed with *pXYLA,XKS1* (YKB2667). Biomass (*closed circles*), glucose (*open squares*), ethanol (*open circles*), glycerol (*open triangles*), and acetate (*closed triangles*). The batches were terminated when no further changes in OD was observed. Each data point represents the mean±SD from duplicated experiments.
