# Supplementary material for: Chemical and Synthetic Genetic Array Analysis Identifies Genes that Suppress Xylose Utilization and Fermentation in Saccharomyces cerevisiae
Source: G3 (Bethesda). 2011 Sep 1;1(4):247–58. doi: 10.1534/g3.111.000695 (PMC3276145; doi:10.1534/g3.111.000695)
Supplement: Supporting Information [file supp_1.4.247_TableS2.pdf]

Table S2 Double mutants do not display additive improvement in xylose utilization.

Strain	Glucose (g/L consumed)	Improvement Glucose (compared to WT)	Xylose (g/L consumed)	Improvement Xylose (compared to WT)
WT	8±0.05	N/A	0.5±0.04	N/A
alp1Δ isc1Δ	7.1±0.015	-11.25	6.5±0.05	1200
alp1Δ rpl20bΔ	7.2±0.02	-10	6.7±0.08	1240
isc1Δ rpl20bΔ	7±0.06	-12.5	6.6±0.074	1216
bud21Δ alp1Δ	8.1±0.09	1.25	6.2±0.06	1140
bud21Δ isc1Δ	8.4±0.08	5	7.15±0.15	1330
bud21Δ rpl20bΔ	8.3±0.1	3.75	6.5±0.06	1200

Strain	Glucose (g/L consumed)	Improvement Glucose (compared to WT +p*XYLA,XKS1)*	Xylose (g/L consumed)	Improvement Xylose (compared to WT +p*XYLA,XKS1*)
WT +*pXYLA,XKS1*	8.1±0.02	N/A	5.8±0.09	N/A
alp1Δ isc1Δ +p*XYLA,XKS1*	8.6±0.08	6.2	7.2±0.02	24.1
alp1Δ rpl20bΔ +p*XYLA,XKS1*	8.2±0.074	1.23	7±0.03	20.7
isc1Δ rpl20bΔ +p*XYLA,XKS1*	8.3±0.08	2.5	6.2±0.02	6.9
bud21Δ alp1Δ +p*XYLA,XKS1*	8.0±0.08	-1.23	7.2±0.03	24.16
bud21Δ isc1Δ +p*XYLA,XKS1*	8.4±0.09	3.7	7.4±0.04	27.6
bud21Δ rpl20bΔ +p*XYLA,XKS1*	8.2±0.1	1.23	6.8±0.1	17.26

Analysis of the consumption of glucose and xylose of the double mutants of the suppressors. Strains tested were Wild-type (YKB731), *alp1Δ isc1Δ* (YKB2534), *alp1Δ rpl20bΔ* (YKB2535), *isc1Δ rpl20bΔ* (YKB2536), *bud21Δ alp1Δ* (YKB2537), *bud21Δ isc1Δ* (YKB2538), *bud21Δ rpl20bΔ* (YKB2539) without (top table) and with *pXYLA,XKS1* (bottom table). Growth cultures were started in either 13% glucose or xylsoe media, pitched with 1.5 x 10^7 cells/ml and carried out at 30° with for 168 hours. The calculation for g/L consumed of sugars corresponds to the concentrations of sugars observed at the end of the batches compared to the starting amount.
